# Self-Reported Food Handling Behaviors of U.S. Consumers While Grilling Poultry and Meat †

**DOI:** 10.3390/foods14071141

**Published:** 2025-03-25

**Authors:** Taylor Terry, Edgar Chambers, Sandria Godwin, Edgar Chambers

**Affiliations:** 1 Center for Sensory Analysis and Consumer Behavior, Kansas State University, Manhattan, KS 66506, USA; 2 Department of Family and Consumer Sciences, Tennessee State University, Nashville, TN 37209, USA; 3 Independent Researcher, 3125 Beavercreek Dr., Cape Girardeau, MO 63701, USA

**Keywords:** food safety, survey, contamination, foodborne illness

## Abstract

Every year, 48 million people in the United States are affected by foodborne illnesses. Twenty-five percent of reported foodborne illnesses are due to unsafe food behaviors in the home. Consumers are grilling more frequently throughout the year; however, little is known about their food handling practices when doing so. Therefore, it is important to investigate the food safety practices of consumers when using an outdoor grill to prepare meat and poultry. A nationwide web survey of consumers who grill outdoors (*n* = 1024) was conducted to approximate the percentage of consumers following recommended food handling practices while grilling. Results showed that there was low adherence to not washing meat/poultry, using separate or clean utensils after flipping or turning the meat or poultry on the grill, or using a thermometer to determine doneness. Sixty-three percent of respondents washed meat or poultry before grilling. Only 22% reported washing utensils after turning or moving the poultry or meat on the grill, which can cause potential cross- or re-contamination. Consumers used many techniques to determine the doneness of meat/poultry, but only 25.6% used a thermometer. The results differed by some demographic characteristics. Women and respondents who grilled poultry versus meat were more likely to follow safe food handling practices. This survey indicates that food safety educators should focus strongly on cross- or re-contamination and thermometer use when designing food safety content for the grilling population.

## 1. Introduction

Foodborne illness is a major public health concern in the United States (U.S.). Although preventable, there are an estimated 48 million cases of foodborne illnesses each year [1]. Foodborne infections are responsible for 128,000 hospitalizations and 3000 deaths, costing the U.S. approximately USD 15.5 million annually [1]. Recent data show that reported cases of foodborne illness are increasing in the U.S. [2], although some of that increase may come from improved data collection. Nearly 25% of the reported cases of foodborne illnesses are due to consumers using unsafe food handling practices in the home [3]. In 2023, U.S. consumer confidence was lower than in recent years, with nearly half of consumers being concerned with “bacterial” contamination of foods [4]. This reduction in consumer confidence in food safety was true for nearly every age, gender, income, and racial/ethnic group, except Generation Z, the youngest consumers studied. Many consumers underestimate the risk of foodborne illnesses; therefore, they may not take the appropriate preventative measures to reduce the risk of foodborne illness [5]. It is widely understood that consumer behavior that results in inappropriate storage, preparation, and cross-contamination of food can result in foodborne illness regardless of the safety of the food when purchased [6]. Improper hand washing, cross-contamination, undercooking meat and poultry, and inadequate storage of foods have been reported by consumers in previous food safety studies [7,8,9,10]. A lack of awareness and knowledge about food safety accounts for some portion of unsafe food handling practices based on recent data published by the U.S. Department of Agriculture (USDA) that showed some changes, but persistent problems, in consumer behavior related to hand washing, thermometer use, and washing/rinsing foods [11,12,13]. To educate consumers about safe food handling practices, the Partnership for Food Safety Education created the FightBAC! campaign. FightBAC! consists of four core recommendations for consumers to follow: Clean, “Wash hands and surfaces often”; Separate, “Don’t cross-contaminate”; Cook, “Cook to the safe internal temperature”; Chill, “Refrigerate promptly” [14].

Although numerous studies have evaluated food safety behaviors in a domestic home kitchen, consumer food safety behaviors in an outside environment, such as when using an outside grill, have not been thoroughly explored. In 2018, a study reported that the use of a food thermometer when grilling during tailgating at a football game was less than 40% before a food safety intervention raised that to almost 60% [15]. Other recent research also shows problems with thermometer use during grilling [12]. Research shows that approximately 70% of households in the United States own a grill or a smoker [16] and that consumers grill year-round. With more consumers grilling more frequently, research must be conducted to understand what food safety practices are being used by consumers while grilling. A national telephone survey from the U.S. Food and Drug Administration found that less than 5% of consumers who use a grill for cooking meat used the same plate to remove food from the grill as what was used to carry the food to the grill [17]. However, other studies have shown that positive food safety behaviors are reported at considerably higher percentages than actually occur based on observational studies [18,19]. That does not negate the value of surveys but does raise concerns that percentages may be skewed positively. However, surveys often do portray issues with food safety. Recent surveys showed that cross-contamination and the lack of the use of a thermometer were primary food safety concerns when consumers use an outdoor grill [12,15].

The objective of this study was to assess the food handling practices of consumers when grilling meat or poultry outdoors. The results of this research can demonstrate what food handling practices consumers are or are not following when preparing meat or poultry in an outside environment and potentially lead to improved consumer messaging.

## 2. Materials and Methods

A nationwide, web-based survey was conducted to collect information on the food-handling behaviors of consumers who grill meat and poultry. The questionnaire was administered by Qualtrics (Provo, UT, USA), a private, web-based research software company.

### 2.1. Sample

The survey was distributed nationwide through an online panel using Qualtrics Research Software 2025. Panel members aged 18 years and older who had prepared meat or poultry using an outdoor grill within the past summer and fall months were granted access to complete the survey. A 70/30 ratio of men and women, respectively, was used to reflect the population of outdoor grilling consumers in the U.S. [16]. A minimum of half of the respondents were required to have prepared poultry on an outdoor grill to assess if differences occur in the food handling behaviors when grilling poultry versus meat. The survey was equally distributed to the four regions of the United States assigned by the U.S. Census Bureau to be representative of the U.S. population (U.S. Census Bureau 2015).

### 2.2. Questionnaire

The concept of the questionnaire was based on the four core practices of food safety from the Partnership for Food Safety Education: Clean, Separate, Cook, and Chill [14]. The specific questions used were determined based on prior surveys of food safety behaviors [20,21,22,23] and in-person qualitative interviews with consumers while grilling chicken, beef, or pork. The interviews, conducted by professionally trained moderators, occurred in three cities (Scottsdale AZ—western USA, Manhattan, KS—Midwestern USA, and Nashville, TN—Southeastern USA). The cities were chosen because they represent various parts of the USA and there was access to trained interviewers and a database of consumers willing to be interviewed while grilling. The interviews included watching and talking with consumers as they prepared meats for cooking (typically in their indoor kitchen), during transport to grills (typically on back porches or outdoors), during grilling, and transport to the table for eating. In the in-person interviews, consumers included both genders, a range of ages, and various ethnic backgrounds.

The questionnaire consisted of 50 questions separated into four different sections to assess the food handling behaviors of respondents who prepared meat (beef, pork, lamb, and veal) or poultry (chicken, turkey, duck, and other birds) on an outdoor grill. The first section asked respondents about their grilling environment, the type of poultry/meat that was grilled, and how the meat/poultry was prepared before grilling. This section also asked respondents how the meat or poultry was handled during steps of preparation, such as opening the package, seasoning or marinating, and moving on the grill. The second section was composed of questions about respondents’ cleaning habits while preparing meat/poultry for grilling and grilling the meat/poultry. Respondents were asked if and how the grill grates were cleaned and how hands, utensils, and dishes were cleaned immediately after they were used to handle the raw meat/poultry. Questions concerning the determination of doneness, satisfaction of the grilled meat/poultry, and handling of leftovers were asked in the third section. The last section was comprised of demographic questions, which included gender, ethnicity, education, and household income, in addition to grill use, frequencies, and perceived skills.

Respondents were asked to answer the questions based on the last time they prepared meat or poultry on an outdoor grill. Questionnaires were self-administered online. The survey items were multiple choice or forced choice to ensure that respondents answered all questions within the survey. The survey was pretested for time estimation, question comprehension, and clarity (*n* = 50). Data collected from the pilot testing were not included in the final data. The survey took an average of 12 min to complete.

### 2.3. Data Analysis

Data were analyzed using XLSTAT software (version 2023.1, Addinsoft, Inc., New York, NY, USA) for Microsoft Excel. Descriptive statistical analyses were conducted for all items in the survey. Cross tabulations and analysis of variance were performed to determine if differences between food-handling behaviors and demographic characteristics exist. For analyses, statistically significant (*p* ≤ 0.05) differences were determined.

## 3. Results

### 3.1. Demographics

In total, data were collected from 1024 respondents. Table 1 provides the demographic characteristics of the respondents. The sample included 743 males (72.6%) and 281 females (27.4%). The majority of respondents were Caucasian (77.4%), between 36 and 65 years of age (49.9%), and had at least some college education (74.9%). Annual household income was distributed with 21.6% of respondents earning less than USD 25,000, 26.5% earning between USD 25,000 and USD 49,000, 33.2% earning between USD 50,000 and USD 99,000, and 18.7% earning over USD 100,000 per year. Over half of the respondents (51.4%) did all the purchasing of groceries in their households.

### 3.2. Types of Meat Grilled

Table 2 shows the type of meat (*n* = 520) or poultry (*n* = 504) that consumers prepared the last time they grilled. Chicken (46.1%) and beef (42.1%) accounted for most of the samples. For beef, steaks (46.2%) and ground beef patties (29.9%) were the specific meats that were prepared on a grill. Chicken parts such as breasts, thighs, wings, and legs (89.6%) were the chicken cuts mostly prepared by respondents the last time they grilled.

### 3.3. Grilling Information

Most respondents (66.4%) used their grill all year long (Table 3). During the grilling season for their area, most respondents (81.6%) grill a few times a month or more. Throughout the off-season, when people do not typically grill, 28.4% of respondents still grill a few times a month, and 25.7% only grill once every two to three months. The majority of respondents (85.4%) reported grilling at their primary residence the last time they grilled using a liquid propane gas grill (41.5%) or charcoal grill (40%). When asked to rate their grilling skills, most respondents rated themselves as average or better (74.2%).

### 3.4. Food Safety Survey Results: Clean

Most consumers washed intact cuts of poultry/meat before grilling (Table 4 and Table 5). Only 23% and 46.2% of consumers did not wash those cuts of poultry or meat, respectively, during preparation for grilling. Consumers who grilled meat were less likely to wash their meat than consumers who grilled poultry. As seen in Table 4, respondents who (a) grilled meat who were African American or Hispanic, (b) from 18 to 35 years of age, (c) had a graduate, professional, or doctorate degree, or (d) had a household income of USD 100,000 or more were more likely to wash the meat.

Eighty-one percent of respondents who grilled poultry cleaned the dishes and utensils used to prepare the raw poultry by washing them with soap, bleach, or a disinfectant and water, or by putting them in the dishwasher to be cleaned before using those dishes for other foods. Of those who grilled meat, 79.5% of the respondents reported to have properly cleaned the dishes and utensils before further use. For both meat and poultry, about 19% of respondents rinsed or continued to use the dishes to prepare other foods, without washing them with soap and water first. Respondents who were 36 years of age or older were more likely to properly clean the dishes or utensils after using them to handle raw poultry. Consumers who were (a) male, (b) between the ages of 18 and 35, (c) had an advanced degree, or (d) earned USD 100,000 or more annually were more likely not to wash utensils or dishes after they were used to handle raw meat. Respondents were asked to report how they handled the raw meat and poultry, such as with bare hands, gloves, or utensils, during particular situations while grilling. More respondents used their bare hands to open packaging (73.6%) and to season or marinate the meat/poultry (58.8%). However, more respondents used a utensil to put the meat/poultry on the grill (58.8%), turn or move the meat/poultry on the grill (81.2%), and remove the meat/poultry from the grill (84.4%).

Most respondents reported cleaning their bare hands, gloves, or utensils immediately after opening the packaging (60.3%) and seasoning or marinating the raw meat/poultry (53.6%). Only 21% and 22.3% of respondents, respectively, cleaned their hands, gloves, or utensils immediately after moving the poultry or meat on the grill. It is not typical for consumers in the USA to have sinks near their outdoor grills, which may make it difficult for them to clean their hands and utensils after starting the grilling process.

Eighty-six percent of respondents cleaned the grates of the grill before grilling, and 61% of respondents cleaned the grates after grilling. Of those respondents, a large majority (95%) used a grill brush or a sturdy utensil on the grill during the cleaning process. Only 44.2% of the respondents cleaned the outside of the grill such as the side shelves and the handles after grilling.

### 3.5. Food Safety Results: Separate

While grocery shopping, 57.4% of respondents used the plastic bags found in the meat section of the store to separate meat and poultry from other foods (Table 6 and Table 7). The majority of the respondents (81.6%) reported that the meat or poultry was bagged separately from other food at the checkout counter.

Of the consumers who thawed their poultry in the refrigerator (*n* = 118), 26.3% of consumers indicated they placed the poultry on the top shelf, 32.2% on the middle shelf, 39.1% on the bottom shelf, and 3.4% stored the poultry in a drawer. Respondents who had a household income of from USD 25,000 to USD 49,000 were more likely to store the poultry on the bottom shelf. For consumers who thawed meat in the refrigerator (*n* = 90), 22.2% placed the meat on the top shelf, 50% on the middle shelf, 25.7% on the bottom shelf, and 2.2% stored the meat in a drawer. Respondents who were younger than sixty-five were more likely to place the meat on the bottom shelf while thawing in the refrigerator.

Out of 644 respondents who used a cutting board or surface to prepare the raw poultry or meat, 73.2% and 66.9% of the respondents, respectively, cleaned the cutting board or surface by either washing with soap, bleach, or disinfectant or by putting it in the dishwasher to be cleaned before using it again to prepare other food or storing. Twenty-seven percent of respondents who grilled poultry and 31.5% who grilled meat rinsed or wiped the cutting board or surface before further use or storing it or they continued to use the cutting board or surface without rinsing or washing it to prepare other food. Respondents who were (a) male, (b) from 18 to 25 years of age, (c) had an annual household income of USD 100,000 or more, (d) had a Bachelor’s, graduate, professional, or doctorate degree, or (e) had less than average grilling skills were less likely to wash the cutting board used for raw poultry before preparing the next food item or storing. Similarly, those who were (a) male, (b) from 18 to 35 years old, or (c) had a household income of USD 100,000 or more were less likely to properly clean the cutting board used to prepare meat before storing or using it on another food item.

Ninety-one percent and 86.9%, respectively, transferred the cooked meat or poultry from the outdoor grill to the kitchen using a clean plate, pan, or dish. Female respondents were more likely to use a clean plate to transfer the meat from the grill to the kitchen. For both poultry and meat, less than 11% of the consumers reported that they transferred the cooked meat using the same plate, pan, or dish that was used for the raw meat or poultry, which can pose a risk of contaminating the cooked meat or poultry.

### 3.6. Food Safety Results: Cook

Of the respondents who marinated raw meat or poultry and reused the marinade (*n* = 70), only 43.6% (*n* = 17) who grilled poultry, and 22.6% (*n* = 7) who grilled meat, boiled the marinade that was used on raw poultry/meat before using it on cooked food items (Table 8 and Table 9). Fifty-six percent of respondents who grilled poultry and 77.4% who grilled meat either heated the marinade, but not to a boil, or did not reheat the marinade at all before using it on cooked foods. Respondents who grilled meat and were between the ages of 18 and 35 were more likely to boil the marinade that was used on raw meat before using the marinade on cooked foods. Respondents who grilled poultry and had a graduate, professional, or doctorate degree were more likely to boil the marinade. Overall, respondents used a variety of techniques to determine the doneness of the meat and poultry. The most used techniques among the respondents to determine the doneness of the meat and poultry were cutting the meat open (42.3% and 48.8%) and looking at the color of the meat (42.1% and 38.7). Twenty-eight percent of the respondents who grilled meat touched the meat for firmness. Twenty-eight percent of the respondents who grilled poultry checked that the juices ran clear. Twenty-three percent and 28.9%, respectively, used a thermometer to check the doneness of the meat or poultry the last time they grilled. Consumers who were younger than 65 and grilled poultry, had an Associate’s degree, a household income of USD 100,000, or had a less than average grilling skill were less likely to use a thermometer to check the doneness of the poultry. Consumers who grilled meat and were males, aged 18–35, Hispanic, had a graduate, professional, or doctoral degree, or a household income of USD 100,000 or more were less likely to check the doneness of meat by using a thermometer.

Half of the respondents (50.1%) reported checking the doneness of only a few pieces of the meat/poultry, while 22.3% checked every piece of the meat/poultry to ensure that all were cooked to the same level of doneness. The majority of the respondents (92.5%) were content with the doneness of each piece of meat/poultry, but 2.6% reported that some pieces were undercooked and 4.2% of respondents reported that there were overcooked pieces.

### 3.7. Food Safety Results: Chill

The Partnership for Food Safety Education (PFSE) recommends that consumers defrost foods in the refrigerator, in cold water that is changed often, or in the microwave oven to keep foods at a safe temperature (PFSE 2016). Only 53% and 55.8% of respondents properly thawed their meat or poultry respectively the last time they grilled (Table 10 and Table 11). For respondents who incorrectly thawed meat, 38.3% thawed the meat on a countertop, 4.2% thawed meat in cold water that was rarely/never changed, and 4.6% thawed meat in warm or hot water. Respondents who grilled meat and were between 18 and 35 were less likely to thaw meat correctly. For respondents who incorrectly thawed the poultry, 30.3% of respondents thawed poultry on a countertop, 2.2% thawed poultry in cold water that was rarely/never changed, and 11.0% thawed poultry in warm or hot water. Respondents who grilled poultry and were (a) 65 or older, (b) had a household income of from USD 50,000 to USD 99,000, or (c) had average or better than average grilling skills were more likely to thaw the poultry correctly.

When marinating and seasoning the raw poultry, 43.8% of the respondents let the poultry rest or marinate in the refrigerator, while 28.1% let the poultry rest on the countertop. Respondents who were (a) between 18 and 35 years of age, (b) had a high school diploma or less or a graduate, professional, or doctorate degree, or (c) had less than less than average grilling skills were less likely to correctly rest or marinate the poultry in the refrigerator. Thirty-two percent of the respondents let the meat marinate in the refrigerator and 34.7% let the meat marinate on the countertop. Respondents who had a graduate, professional, or doctorate degree were more likely to let the meat rest or marinate on the countertop.

The USDA recommends that leftovers be stored in the refrigerator or freezer within 2 h of removing from the heat source and eaten within 3–4 days to reduce bacterial growth [24]. After grilling the poultry and meat, 81.1% and 73.5% of respondents who had leftovers, respectively, left them sitting at room temperature for no more than 2 h. For both, meat and poultry, less than 1% let their leftovers sit at room temperature for two hours or more.

## 4. Discussion

This survey assessed the food handling practices of consumers when grilling meat or poultry. Demographic differences among consumer food handling practices were identified. Consistent with other food safety surveys, the findings of this study show that risky food handling behaviors are prevalent among consumers when grilling. Foodborne illness is a serious issue with more than 1 million people getting sick in the USA each year from various foodborne pathogens including *Campylobacter jejuni*, *Clostridium perfringens*, *Escherichia coli* O157:H7, and *Salmonella* (multiple types) found in undercooked meat and poultry or causing cross-contamination during handling [25]. Maintaining hygiene standards, proper handling to prevent cross-contamination, thorough cooking to appropriate temperatures measured by a thermometer properly inserted into the product, and appropriate storage after cooking are the primary ways in which consumer control of foodborne illness can be accomplished [14].

Despite most respondents reporting washing their hands with soap and water after handling raw meat or poultry, it is uncertain whether they followed the proper procedures for hand washing: washing hands with warm water and soap for at least 20 s. Similarly, most respondents reported following safe practices when using cutting boards, dishes, and utensils to prepare raw meat or poultry, but it is unknown if the items were washed adequately. When opening a package with bare hands, 20.1% of respondents who grilled meat and 15.1% who grilled poultry reported they did not clean or wash their hands with soap and water. Previous research has shown that the outside of meat or poultry packages can be a vehicle for cross-contamination [26,27,28,29,30]. Several authors found bacteria, such as *Campylobacter* and *Salmonella* strains, were present on the external packaging of raw meats [26,28]. Therefore, it is recommended that consumers wash their hands thoroughly after touching a package of meat or use a bag or gloves to pick up raw meat and poultry to reduce the spread of bacteria [27,28].

Over 43% and 78% of respondents reported washing raw meat or poultry, respectively, before grilling. The USDA recommends that consumers do not wash raw meat or poultry to prevent splashing of contaminated water onto other foods, utensils, or kitchen surfaces based on multiple studies [31]. Rinsing meat and poultry can increase the likelihood of harmful bacteria being spread throughout the kitchen [32,33,34]. Research [35] has shown that, when washing poultry, droplets from the contaminated water could travel 70 cm away from the sink or site of washing. Washing or rinsing raw meat or poultry does not remove pathogens such as *Salmonella* strains; thus, it serves no purpose for food safety [36].

Cross-contamination is at the top of the list of food safety concerns during the grilling season. To prevent cross-contamination, raw foods should always be separated from cooked or ready-to-eat foods [14]. In addition, consumers are advised to store raw foods in a sealed container or plastic bag on the bottom shelf of the refrigerator to prevent juices from leaking and contaminating other foods. Consistent with previous research [21,27], few consumers stored the meat on the bottom shelf of the refrigerator. Although about one-third of respondents stored the meat or poultry to be grilled correctly in the refrigerator, it is not known if the meat or poultry was stored in a sealed container or plastic bag to further reduce the risk of contamination.

Many respondents did not clean the utensils immediately after putting the meat on the grill and moving or flipping the meat/poultry. Using the same utensil, without washing or sanitizing, throughout the grilling process can pose a risk for cross-contamination [14,37]. It is not safe to use the same utensil used to put the raw meat or poultry on the grill to flip the meat. The utensil will be contaminated with bacteria from the raw meat or poultry; therefore, if used again without washing, it could contaminate the cooked portion of the meat or poultry. The USDA recommends that consumers do not use the same utensils for raw and cooked meats and poultry [38,39]. Once a utensil has touched raw meat or poultry, it should be washed with soap before contact with cooked or ready-to-eat foods [14,37]. It is best to wash dishes and utensils in between uses until the meat or poultry has been thoroughly cooked or to use different dishes or utensils to reduce the occurrence of cross-contamination. Of course, a lack of adjacent washing facilities makes this more difficult to do easily, and consumers may simply choose to forego that step because it takes extra effort.

Although most consumers discarded the excess marinade that was used on raw meat and poultry, a few respondents incorrectly reused the marinade by not boiling the marinade to ensure bacteria was killed before using it on cooked foods. The PFSE recommends consumers boil used marinade before applying it to cooked foods to destroy harmful bacteria or to reserve a portion of the unused marinade to use as a sauce [40].

Temperature plays an important role in the safety of raw meats and poultry. To kill any pathogens that may be present, meat and poultry should be cooked thoroughly to a safe internal temperature [41]. Poultry (ground or intact cuts) should be cooked to a safe minimum temperature of 73.9 °C (165 °F), cuts of beef, pork, veal, and lamb cooked to an internal temperature of 62.8 °C (145 °F), and ground meats, such as hamburgers, cooked to 71.1 °C (160 °F). The only way to ensure that meat or poultry has been cooked to a safe minimum internal temperature and that foodborne bacteria have been destroyed is by using a thermometer [7,8,15,20,41,42]. Previous research has shown that few consumers use a thermometer regularly to gauge the doneness of meat and poultry [9,13,21,43]. Only about one-fifth of respondents in this survey used a thermometer the last time they grilled to check if the meat or poultry was done, but it is not known if the meat or poultry was cooked to a safe internal temperature or if the thermometer was used correctly. Most respondents used a variety of techniques other than using a thermometer to check the doneness of the meat or poultry, such as looking at the color of the meat, cutting the meat open, checking that the juices ran clear, touching the meat for firmness, and using previous experience. Using subjective assessments of the doneness of meat and poultry is unsafe. Research has shown that hamburgers can turn brown in the middle before reaching a safe internal temperature [44]. Similarly, another study found that 70% of chicken pieces that were deemed “done” by consumers who visually inspected the chicken were undercooked and had active *Campylobacter jejuni* cells [45]. Another study showed that the visual color of ground chicken patties was dependent on the type of lighting available, such as fluorescent or light-emitting diode (LED) [46].

There were a considerable number of respondents who thawed or marinated meat/poultry on the countertop or in warm or hot water. Neither of these thawing methods is safe, as foods at a temperature between 4 °C and 60 °C (40 °F and 140 °F) can cause bacteria to multiply rapidly [47,48,49]. When handling leftovers, it is important to refrigerate or freeze foods promptly and at the proper temperature. To slow the growth of harmful bacteria, consumers should chill leftovers at a temperature of 4 °C (40 °F) within two hours of sitting at room temperature or within one hour if the leftovers are sitting at temperatures above 32 °C (90 °F) [39]. The majority of respondents refrigerated or froze their leftovers within two hours or less.

There was considerable consistency across many behavioral characteristics that could result in foodborne illness. Many consumers did not follow “best practices” in cleaning, handling, cooking, and storing grilled meat and poultry. However, some demographic segments were less likely than others to follow the recommended food safety practices. Males, those aged 18–35, respondents with graduate or professional degrees, and those with incomes over USD 100,000 were more likely than others to report practicing poor food safety behaviors. Although we did not evaluate the reasons for good or poor practices, we can speculate on potential reasons that these groups were less likely to follow guidelines for safe food handling. Although food shopping and cooking roles are changing, women still do most of the grocery shopping and cooking [50]. Thus, many men may be less likely than women to encounter food safety messaging in grocery stores and on food packaging. Those with higher education, which co-varied with higher income in our study, also reported fewer positive food safety behaviors. Although that group is widely believed to have better health outcomes, food preparation may not be considered a particularly valuable activity that needs “scientific” information. In fact, one study reported that cooking was primarily seen as a creative outlet [51]. In addition, people with a higher level of education (and income in this study) have been shown previously to have less free time than those with lower education levels [52]. Those two facts together could suggest that individuals with higher education and income are less likely to prioritize learning food safety information. Some individuals who were in the lower age group (18–35) may have less exposure to food safety education materials or may prioritize such information at a lower level given the importance of getting an education and starting careers and families.

A prevailing problem identified in this research was not using a thermometer to check doneness, which had been noted in multiple other studies [15,42]. Various reasons for not using thermometers have been given, including the following: no meat or similar thermometer in the home, too much trouble to find and use the thermometer, no need to use a thermometer because “it hasn’t been needed before”, and not needing to use a thermometer when other methods such as visual inspection, time, or touch can be used [20].

All the issues described in this research may be overcome with appropriate health messaging. It is important to note that educational materials targeting health outcomes, in this case, safe food consumption, must be reliable, timely, and provide a unified message [53]. However, those same authors note that educational messaging must also clearly identify the “threat” (foodborne illness) in a way that recognizes and adapts the messaging to account for “levels of preconception and understanding” and the “perceived susceptibility/vulnerability” of the targeted population. No matter the demographic of the target population, be it age, gender, education level, etc., we must remember that the vast majority of consumers are not experts in food safety and need information that provides them with the ability to recognize the “threat” and overcome it.

## 5. Conclusions

This study provides information on the food handling practices of consumers when grilling meat or poultry. Overall, respondents who grilled poultry reported following some safer food handling practices when grilling raw poultry. Women were significantly more likely to follow safe handling practices than men. Based on the statistical differences that were identified between demographics and safe food handling practices, the demographic groups that should be targeted for food safety education are men, young adults, lower-middle-income and higher-income consumers, and consumers with postgraduate degrees. Educational efforts should focus on not washing meat/poultry, cross-contamination prevention when flipping or moving the meat/poultry on the grill, and the importance of thermometer use. To promote safe handling of food while grilling, food safety content could be displayed on the packaging of grilling essentials, such as liquid propane tanks, charcoal bags, meat forks, or on a panel of the grill itself.

## Figures and Tables

**Table 1:** Demographics of respondents (*n* = 1024).

	*n*	%
**Gender**		
Male	743	72.6
Female	281	27.4
**Age**		
18–35	333	32.5
36–64	511	49.9
65+	180	17.6
**Ethnicity**		.
Caucasian	793	77.4
African American	88	8.6
Hispanic	60	5.9
Asian	47	4.6
Native American	17	1.7
Other	19	1.9
**Education**		
Some high school or less	25	2.4
High school graduate or GED	232	22.7
Some college (no degree)	262	25.6
Associate’s degree	112	10.9
Bachelor’s degree	242	23.6
Graduate or professional degree	123	12.0
Doctoral degree	28	2.7
**Household Income**		
Less than USD 25,000	221	21.6
USD 25,000 to USD 49,000	271	26.5
USD 50,000 to USD 99,000	340	33.2
USD 100,000 or more	192	18.8

**Table 2:** Type of raw meat or poultry consumers prepared the last time they grilled outside (*n* = 1024).

	*n*	%
**Meat**	520	50.8
Beef	432	42.2
Pork	73	7.1
Lamb or sheep	11	1.1
Veal	3	0.3
Other meat	1	0.1
**Poultry**	504	49.2
Chicken	472	46.1
Turkey	23	2.2
Duck or other birds	9	0.9

**Table 3:** Grilling area, usage, and skill of the respondents.

	*n*	%
**Grilling Area**		
Grill with no side shelves or surface area to place plates or utensils.	268	26.2
Grill with side shelves or a surface area to place plates or utensils.	694	67.8
Grill with an outdoor kitchen with countertops.	45	4.4
Grill with an outdoor kitchen with countertops and a sink.	17	1.7
**Grill Type**		
Liquid Propane Grill	425	41.5
Natural Gas Grill	133	13.0
Charcoal Grill	410	40.0
Electric Grill	51	5.0
Ceramic Grill	5	0.5
**Year-Round Grill Usage**		
Yes	680	66.4
No	344	33.6
**Grilling Skill**		
Novice/Beginner	36	3.5
Basic skills	160	15.6
Average	401	39.2
Better than average	358	35.0
Expert	69	6.7

**Table 4:** Food handling practices of respondents who grilled meat (*n* = 520).

Safe Food Handling Practices	Did Not Wash or Rinse Intact Meat		Washed Dishes and Utensils Used to Prepare and Handle Raw Meat		Washed Hands, Gloves, or Utensils Immediately After Turning or Moving Meat on the Grill	
	***n***	**%**	***n***	**%**	***n***	**%**
**Gender**						
Male ^1^	149	43.4	238	69.4	72	21.0
Female	91	51.4	160	90.4 *	45	25.4
**Age**						
18–35	60	33.9 *	115	64.8 *	37	20.9
36–64 ^1^	125	49.6	211	83.7	67	26.6
65+	55	60.4	72	79.1	13	14.3
**Ethnicity**						
Caucasian ^1^	211	49.5	329	77.2	96	22.5
African American	9	26.5 *	24	70.6	10	29.4
Hispanic	4	15.4 *	22	84.6	6	23.1
Asian	7	46.7	9	60.0	3	20.0
Native American	7	50.0	11	78.6	2	14.3 *
Other	2	40.0	3	60.0	0	0.0 *
**Education**						
High School or less	57	42.5	103	76.9	33	24.6
Some college	76	54.7	110	79.1	31	22.3
Associate’s degree	27	44.3	53	86.9	12	19.7
Bachelor’s degree ^1^	54	46.6	91	78.5	25	21.6
Graduate/professional/doctorate degree	26	37.1 *	41	58.6 *	16	22.9
**Household Income**						
Less than USD 25,000	53	45.7	93	80.2	30	25.9
USD 25,000 to USD 49,000	60	39.7	118	78.2	31	2.05
USD 50,000 to USD 99,000 ^1^	97	59.5	133	81.6	32	19.6
USD 100,000 or more	30	33.3 *	54	60.0 *	24	23.7
**Grilling Skill**						
Less than average	43	41.8	71	68.9	23	22.3
Average ^1^	94	47.0	160	80.0	46	23.0
Better than average	103	47.5	197	77.0	48	22.1
**Total**	240	46.2	398	76.5	117	22.5

^1^ Reference group (the largest group in each demographic segment). * Significantly different from the reference group *p* < 0.05.

**Table 5:** Clean food handling practices of respondents who grilled poultry (*n* = 504).

Safe Food Handling Practices	Did Not Wash or Rinse Intact Poultry ^a^		Washed Dishes and Utensils Used to Prepare and Handle Raw Poultry		Washed Hands, Gloves, or Utensils Immediately After Turning or Moving Poultry on the Grill	
	***n***	**%**	***n***	**%**	***n***	**%**
**Gender**						
Male ^1^	87	21.8	315	78.8	74	18.5
Female	27	26.0	91	87.5	30	28.8
**Age**						
18–35	40	25.7	109	69.9 *	38	24.4 *
36–64 ^1^	59	22.8	217	83.8	51	19.7
65+	15	16.9	80	89.9	15	16.9
**Ethnicity**						
Caucasian ^1^	85	23.2	298	81.2	70	19.1
African American	7	13.0	47	87.0	15	27.8
Hispanic	13	38.2	24	70.6	4	11.8
Asian	6	18.8	23	71.9	10	31.3
Native American	0	0.0	2	66.7	2	66.7
Other	3	21.4	12	85.7	3	21.4
**Education**						
High School or less	20	16.3	97	78.9	31	25.2
Some college	33	26.8	102	82.9	19	15.4 *
Associate’s degree	15	29.4	44	86.3	9	17.6
Bachelor’s degree ^1^	35	27.8	103	83.7	25	20.3
Graduate/professional/doctorate	11	13.6	60	74.1	20	24.7 ^a^
**Household Income**						
Less than USD 25,000	21	20.0	88	83.8	24	22.9
USD 25,000 to USD 49,000	26	21.7	98	81.7	22	18.3
USD 50,000 to USD 99,000 ^1^	45	25.4	141	79.7	32	18.1
USD 100,000 or more	22	21.6	79	77.5	26	25.5
**Grilling Skill**						
Less than average	14	15.1	66	71.0	21	22.6
Average ^1^	53	26.4	164	81.6	40	16.9
Better than average	47	22.4	176	83.8	43	20.5
**Total**	114	22.6.	406	80.6	104	20.6

^1^ Reference group (the largest group in each demographic segment). ^a^ Poultry that was not ground. * Significantly different from the reference group *p* < 0.05.

**Table 6:** Separate food handling practices of respondents who grilled meat (*n* = 520).

Safe Food Handling Practices	Stored Meat on the Bottom Shelf of the Refrigerator During Thawing ^a^		Washed Cutting Board Used for Raw Meat Before Preparing Next Food Item or Storing ^b^		Used a Clean Dish to Remove and Transfer Cooked Meat from the Grill	
	***n***	**%**	***n***	**%**	***n***	**%**
**Gender**						
Male ^1^	14	22.6	124	60.5	286	83.4
Female	9	32.1	80	80 *	166	93.8 *
**Age**						
18–35	8	26.7	68	54.4 *	145	81.9
36–64 ^1^	11	28.2	100	72.5	224	88.9
65+	4	19.1 *	36	85.7 *	83	91.2
**Ethnicity**						
Caucasian ^1^	22	28.6	134	68.1	374	87.8
African American	0	0.0	15	65.2	23	67.7
Hispanic	0	0.0	14	70.0	23	88.5
Asian	0	0.0	6	60.0	15	100.0
Native American	0	0.0	4	50.0	13	92.9
Other	1	100.0	1	33.3	4	80.0
**Education**						
High School or less	7	33.3	54	69.2	118	88.1
Some college	5	27.8	52	69.3	122	87.8
Associate’s degree	2	15.4	25	75.8	54	88.5
Bachelor’s degree ^1^	6	26.1	55	75.3	102	87.9
Graduate/professional/doctorate	3	20.0	18	39.1 *	56	80.0
**Household Income**						
Less than USD 25,000	8	44.4	49	74.2	100	96.2
USD 25,000 to USD 49,000	8	34.8	60	68.2	136	90.1
USD 50,000 to USD 99,000 ^1^	4	12.1	66	69.5	139	85.3
USD 100,000 or more	3	18.8	29	51.8	77	85.6
**Grilling Skill**						
Less than average	1	6.7	39	60.9	82	79.6
Average ^1^	8	22.2	80	69.0	178	89.0
Better than average	14	35.9	85	69.0	192	88.5
**Total**	23	25.6	204	68.0	452	86.9

^1^ Reference group (the largest group in each demographic segment). ^a^ Only respondents who thawed meat in the refrigerator were allowed to answer this question (*n* = 90). ^b^ Only respondents who used a cutting board to prepare meat were allowed to this question (*n* = 90). * Significantly different from the reference group *p* < 0.05.

**Table 7:** Separate food handling practices of respondents who grilled poultry (*n* = 504).

Safe Food Handling Practices	Stored Poultry on the Bottom Shelf of the Refrigerator During Thawing ^a^		Washed Cutting Board Used for Raw Poultry Before Preparing Next Food Item or Storing ^b^		Used a Clean Dish to Remove and Transfer Cooked Poultry from the Grill	
	***n***	**%**	***n***	**%**	***n***	**%**
**Gender**						
Male ^1^	37	38.5	188	69.4	360	90.0
Female	8	36.4	60.0	88.2 *	96	92.3
**Age**						
18–35	7	26.9	73	60.8 *	134	85.9
36–64 ^1^	29	27.5	128	77.1	242	93.4
65+	9	29.0	47	88.7 *	80	89.9
**Ethnicity**						
Caucasian ^1^	32	34.8	182	77.5	336	91.6
African American	4	57.1	27	62.8 *	48	88.9
Hispanic	4	57.1	16	64.0	28	82.4
Asian	3	30.0	14	56.0 *	29	90.6
Native American	0	0.0	1	33.3 *	2	66.7
Other	2	100.0	8	100.0 *	13	92.9
**Education**						
High School or less	11	55.0	66	76.7 *	107	87.0
Some college	13	36.1	61	81.3 *	110	89.4
Associate’s degree	6	40.0	60	90.9 *	48	94.1
Bachelor’s degree ^1^	10	43.5	58	67.4	115	91.3
Graduate/professional/doctorate	5	20.8	33	55.9	76	93.8
**Household Income**						
Less than USD 25,000	6	33.3	50	73.5	92	87.6
USD 25,000 to USD 49,000	12	60.0 *	57	73.1	111	92.5
USD 50,000 to USD 99,000 ^1^	21	36.8	93	77.5	157	88.7
USD 100,000 or more	6	36.1	48	65.8 *	96	94.1
**Grilling Skill**						
Less than average	4	26.7	44	60.3 *	80	86.0
Average ^1^	21	41.2	101	78.3	10	89.6
Better than average	20	38.5	103	75.2	196	93.3
**Total**	45	38.1	248	73.2	456	90.5

^1^ Reference group (the largest group in each demographic segment). ^a^ Only respondents who thawed the poultry in the refrigerator were allowed to answer this question (*n* = 180). ^b^ Only respondents who used a cutting board to prepare the poultry were allowed to this question (*n* = 339). * Significantly different from the reference group *p* < 0.05.

**Table 8:** Cook food handling practices of respondents who grilled meat (*n* = 520).

Safe Food Handling Practices	Boiled Marinade Used on Raw Meat Before Using It on Cooked Foods ^a^		Used a Thermometer to Determine Doneness of Meat	
	*n*	%	*n*	%
**Gender**				
Male ^1^	4	18.2	69	20.1
Female	3	33.3	50	28.3 *
**Age**				
18–35	6	28.6 *	35	19.8 *
36–64 ^1^	1	14.3	62	24.6
65+	0	0.0 *	22	24.3
**Ethnicity**				
Caucasian ^1^	5	23.8	96	22.5
African American	0	0.0	9	26.5
Hispanic	0	0.0	4	15.4 *
Asian	2	66.7	4	26.7
Native American	0	0.0	5	35.7
Other	0	0.0	1	20.0
**Education**				
High School or less	4	44.4	33	24.6
Some college	0	0.0	25	18.0
Associate’s degree	0	0.0	18	29.5
Bachelor’s degree ^1^	2	25.0	33	28.5
Graduate/professional/doctorate degree	1	14.3	10	14.3 *
**Household Income**				
Less than USD 25,000	0	0.0	24	20.7
USD 25,000 to USD 49,000	4	36.4	36	23.8
USD 50,000 to USD 99,000 ^1^	1	25.0	46	28.2
USD 100,000 or more	2	25.0	13	14.4 *
**Grilling Skill**				
Less than average	1	25.0	17	16.5
Average ^1^	2	28.6	48	24.0
Better than average	4	20.0	54	24.9
**Total**	7	22.6	119	22.9

^1^ Reference group (the largest group in each demographic segment). ^a^ Only respondents who reused excess marinade that was used on raw meat were allowed to answer this question (*n* = 31). * Significantly different from the reference group *p* < 0.05.

**Table 9:** Cook food handling practices of respondents who grilled poultry (*n* = 504).

Safe Food Handling Practices	Boiled Marinade Used on Raw Poultry Before Using it on Cooked Foods ^a^		Used a Thermometer to Determine Doneness of Poultry	
	*n*	%	*n*	%
**Gender**				
Male ^1^	14	41.2	117	29.3
Female	3	60.0	29	27.9
**Age**				
18–35	10	47.6	31	19.9
36–64 ^1^	7	46.7	76	29.3
65+	0	0.0	39	43.8 *
**Ethnicity**				
Caucasian ^1^	10	47.6	113	30.8
African American	1	11.1	14	25.9
Hispanic	2	50.0	5	14.7 *
Asian	4	100.0	8	25.0
Native American	0	0.0	1	33.3
Other	0	0.0	5	35.7
**Education**				
High School or less	4	40.0	30	24.4
Some college	1	16.8 *	40	32.5
Associate’s degree	0	0.0 *	25	49.0 *
Bachelor’s degree ^1^	4	44.4	30	23.8
Graduate/professional/doctorate degree	8	80.0 *	21	25.9
**Household Income**				
Less than USD 25,000	4	36.4	37	35.2
USD 25,000 to USD 49,000	3	27.3	36	30.0
USD 50,000 to USD 99,000 ^1^	1	20.0	50	28.3
USD 100,000 or more	9	75.0	23	22.6 *
**Grilling Skill**				
Less than average	6	40.0	18	19.4
Average ^1^	5	50.0	60	29.9
Better than average	6	42.9	68	32.4
**Total**	17	43.6	146	29.0

^1^ Reference group (the largest group in each demographic segment). ^a^ Only respondents who reused excess marinade that was used on raw poultry were allowed to answer this question (*n* = 39). * Significantly different from the reference group *p* < 0.05.

**Table 10:** Chill food handling practices of respondents who grilled meat (*n* = 520).

Safe Food Handling Practices	Thawed Meat in the Refrigerator, Microwave, or with Cold Water That Was Changed Often ^a^		Marinated/Rested Meat in the Refrigerator ^b^		Refrigerated or Froze Leftovers Within 2 h	
	*n*	%	*n*	%	*n*	%
**Gender**						
Male ^1^	78	52.0	96	32.8	248	72.3
Female	36	53.7	49	30.8	134	75.7
**Age**						
18–35	40	46.0 *	42	24.9	134	75.7
36–64 ^1^	51	56.7	82	37.3	185	73.4
65+	23	57.5	21	33.3	63	69.2 *
**Ethnicity**						
Caucasian ^1^	95	54.6	115	31.6	315	73.9
African American	7	43.8	15	44.1	25	73.5
Hispanic	6	46.2	10	41.8	19	73.1
Asian	4	57.1	2	14.3	9	60.0 *
Native American	1	33.3	2	18.2	10	71.4
Other	1	25.0	1	20.0	4	80.0
**Education**						
High School or less	28	50.0	41	35.7	103	76.9
Some college	23	43.4	31	25.6	103	74.1
Associate’s degree	16	64.0	23	41.8	38	62.3
Bachelor’s degree ^1^	28	60.9	37	37.0	87	75.0
Graduate/professional/doctorate degree	19	51.4	13	21.3 *	51	72.9
**Household Income**						
Less than USD 25,000	27	60.0	29	30.5	84	72.4
USD 25,000 to USD 49,000	30	48.4	49	36.0	112	74.2
USD 50,000 to USD 99,000 ^1^	37	53.6	40	28.0	118	72.4
USD 100,000 or more	20	48.8	27	34.6	68	75.6
**Grilling Skill**						
Less than average	18	39.1	28	31.5	79	76.7
Average ^1^	47	53.4	58	34.3	150	75.0
Better than average	49	59.0	59	30.4	153	70.5 *
**Total**	114	52.5	145	32.1	382	73.5

^1^ Reference group (the largest group in each demographic segment). ^a^ Only respondents who thawed meat were allowed to answer this question (*n* = 217). ^b^ Only respondents who marinated or seasoned the meat were allowed to answer this question (*n* = 452). * Significantly different from the reference group *p* < 0.05.

**Table 11:** Chill food handling practices of respondents who grilled poultry (*n* = 504).

Safe Food Handling Practices	Thawed Poultry in the Refrigerator, Microwave, or with Cold Water That Was Changed Often ^a^		Marinated/Rested Poultry in the Refrigerator ^b^		Refrigerated or Froze Leftovers Within 2 h	
	*n*	%	*n*	%	*n*	%
**Gender**						
Male ^1^	125	55.6	145	41.7	329	82.3
Female	28	57.1	50	51.6	83	79.8
**Age**						
18–35	37	42.1 *	51	35.2 *	136	87.2
36–64 ^1^	80	58.4	109	47.6	207	79.9
65+	36	73.5 *	36	49.3	69	77.5
**Ethnicity**						
Caucasian ^1^	112	58.7	135	42.7	302	82.3
African American	14	46.7	28	52.8	43	79.6
Hispanic	10	58.8	14	46.7	28	82.4
Asian	13	56.5	13	41.9	26	81.3
Native American	0	0.0	1	50.0	2	66.7 *
Other	4	44.4	4	30.8	11	78.6
**Education**						
High School or less	32	47.1	37	35.6	103	83.7
Some college	45	69.2	54	49.5	89	72.4
Associate’s degree	19	67.9	25	54.4	36	70.6 *
Bachelor’s degree ^1^	28	48.3	53	47.3	112	88.9
Graduate, professional or doctorate degree	29	52.7	26	35.1 *	72	88.9
**Household Income**						
Less than USD 25,000	28	50.9 *	38	42.2	81	77.1
USD 25,000 to USD 49,000	30	51.0 *	55	52.4	98	81.7
USD 50,000 to USD 99,000 ^1^	65	63.1	62	39.5	144	81.4
USD 100,000 or more	30	52.6 *	40	43.0	89	87.3
**Grilling Skill**						
Less than average	19	36.5 *	34	19.5 *	79	85.0
Average ^1^	66	60.6	73	34.5	160	79.6
Better than average	68	60.2	88	25.9	173	82.4
**Total**	153	55.8	195	43.8	412	81.8

^1^ Reference group (the largest group in each demographic segment). ^a^ Only respondents who thawed poultry were allowed to answer this question (*n* = 274). ^b^ Only respondents who marinated or seasoned the poultry were allowed to answer this question (*n* = 445). * Significantly different from the reference group *p* < 0.05.

## Data Availability

The original contributions presented in this study are included in the article; further inquiries can be directed to the corresponding author.
